# Repeat Cardiovascular Risk Assessment after Four Years: Is There Improvement in Risk Prediction?

**DOI:** 10.1371/journal.pone.0147417

**Published:** 2016-02-19

**Authors:** Parinya Chamnan, Rebecca K. Simmons, Stephen J. Sharp, Kay-Tee Khaw, Nicholas J. Wareham, Simon J. Griffin

**Affiliations:** 1 MRC Epidemiology Unit, Institute of Metabolic Science, University of Cambridge, Cambridge, United Kingdom; 2 Cardio-Metabolic Research Group, Department of Social Medicine, Sanpasitthiprasong Hospital, Ubon Ratchathani, Thailand; 3 Clinical Gerontology Unit, University of Cambridge School of Clinical Medicine, Addenbrooke’s Hospital, Cambridge, United Kingdom; Hospital de Clínicas de Porto Alegre, BRAZIL

## Abstract

**Background:**

Framingham risk equations are widely used to predict cardiovascular disease based on health information from a single time point. Little is known regarding use of information from repeat risk assessments and temporal change in estimated cardiovascular risk for prediction of future cardiovascular events. This study was aimed to compare the discrimination and risk reclassification of approaches using estimated cardiovascular risk at single and repeat risk assessments

**Methods:**

Using data on 12,197 individuals enrolled in EPIC-Norfolk cohort, with 12 years of follow-up, we examined rates of cardiovascular events by levels of estimated absolute risk (Framingham risk score) at the first and second health examination four years later. We calculated the area under the receiver operating characteristic curve (aROC) and risk reclassification, comparing approaches using information from single and repeat risk assessments (i.e., estimated risk at different time points).

**Results:**

The mean Framingham risk score increased from 15.5% to 17.5% over a mean of 3.7 years from the first to second health examination. Individuals with high estimated risk (≥20%) at both health examinations had considerably higher rates of cardiovascular events than those who remained in the lowest risk category (<10%) in both health examinations (34.0 [95%CI 31.7–36.6] and 2.7 [2.2–3.3] per 1,000 person-years respectively). Using information from the most up-to-date risk assessment resulted in a small non-significant change in risk classification over the previous risk assessment (net reclassification improvement of -4.8%, p>0.05). Using information from both risk assessments slightly improved discrimination compared to information from a single risk assessment (aROC 0.76 and 0.75 respectively, p<0.001).

**Conclusions:**

Using information from repeat risk assessments over a period of four years modestly improved prediction, compared to using data from a single risk assessment. However, this approach did not improve risk classification.

## Introduction

Quantitative assessment of cardiovascular risk forms part of strategies for prevention of cardiovascular disease (CVD) in many countries.[1,2] National guidelines suggest that multivariate risk assessment tools, such as the Framingham equations, should be incorporated into a programme of repeat risk assessment in order to identify individuals at high risk who could be targeted for preventive interventions.[3–6]

Risk equations such as the Framingham risk score have been shown to predict cardiovascular disease over the short term reasonably accurately.[7,8] However, there is uncertainty about their predictive ability over the longer term, with some studies suggesting significant predictive discrepancy between short-term and lifetime risk.[9] This raises the possibility of false reassurance in those with low short-term risk but high long-term risk. This concern might be addressed by repeat assessments of short-term cardiovascular risk over time. However, there is a lack of such evidence and the recent guideline by the American College of Cardiology Foundation and American Heart Association stresses that research on the optimal timing to begin risk assessment and repeat risk assessment in asymptomatic low-intermediate risk individuals is needed.[10]

It is unclear whether information from repeat risk assessments (predicted risk at different time points) or changes in predicted risk could improve the predictive ability of cardiovascular risk scores above and beyond information obtained from a single risk assessment. The predictive utility of a cardiovascular risk score could be influenced by changes in the distribution of cardiovascular risk factors and their treatment over time, both within and between populations.[8,11] Although some studies have examined the predictive ability of selected cardiovascular risk factors at different time points,[12] little evidence exists to document the overall predictive ability of risk scores estimated at different time points.[13,14] The recent Editorial by Gaziano and Wilson stresses that the contribution to prediction of repeat risk assessment using standard risk scores merits further study.[14]

We aimed to investigate the association between information from repeat risk assessment (repeat values of the Framingham risk score and change in the score over time) with risk of cardiovascular disease. We examined the added value of (i) repeat values of the Framingham risk score at different time points and (ii) temporal change in the risk score, compared to the score assessed at a single time point, for prediction of cardiovascular disease in a large British cohort.

## Methods

### Study design and population

We used data from the European Prospective Investigation for Cancer (EPIC)-Norfolk study, a large prospective cohort.[15] Between 1993 and 1997, 25,639 men and women, aged 40–74 years, attended a baseline health examination, which included a self-administered health and lifestyle questionnaire, medical history taking, physical examination, and blood testing. Participants completed questionnaires about their personal and family history of disease, medication, and lifestyle factors including diet, physical activity and smoking habits. They were asked whether a physician had ever told them that they had any of the conditions in a list that included diabetes, heart attack, and stroke. Anthropometric and blood pressure measurements, and non-fasting blood samples were also taken at the health assessment. EPIC-Norfolk is similar to a nationally representative sample for anthropometric indices, blood pressure and serum lipids, albeit with a lower prevalence of cigarette smoking.[15]

Participants were invited to attend a second health assessment after four years (1998–2001), at which identical measurements were taken,[16] and 15,028 participants (59%) attended. There was no formal quantitative cardiovascular risk assessment at either health examination.

**Fig 1:**
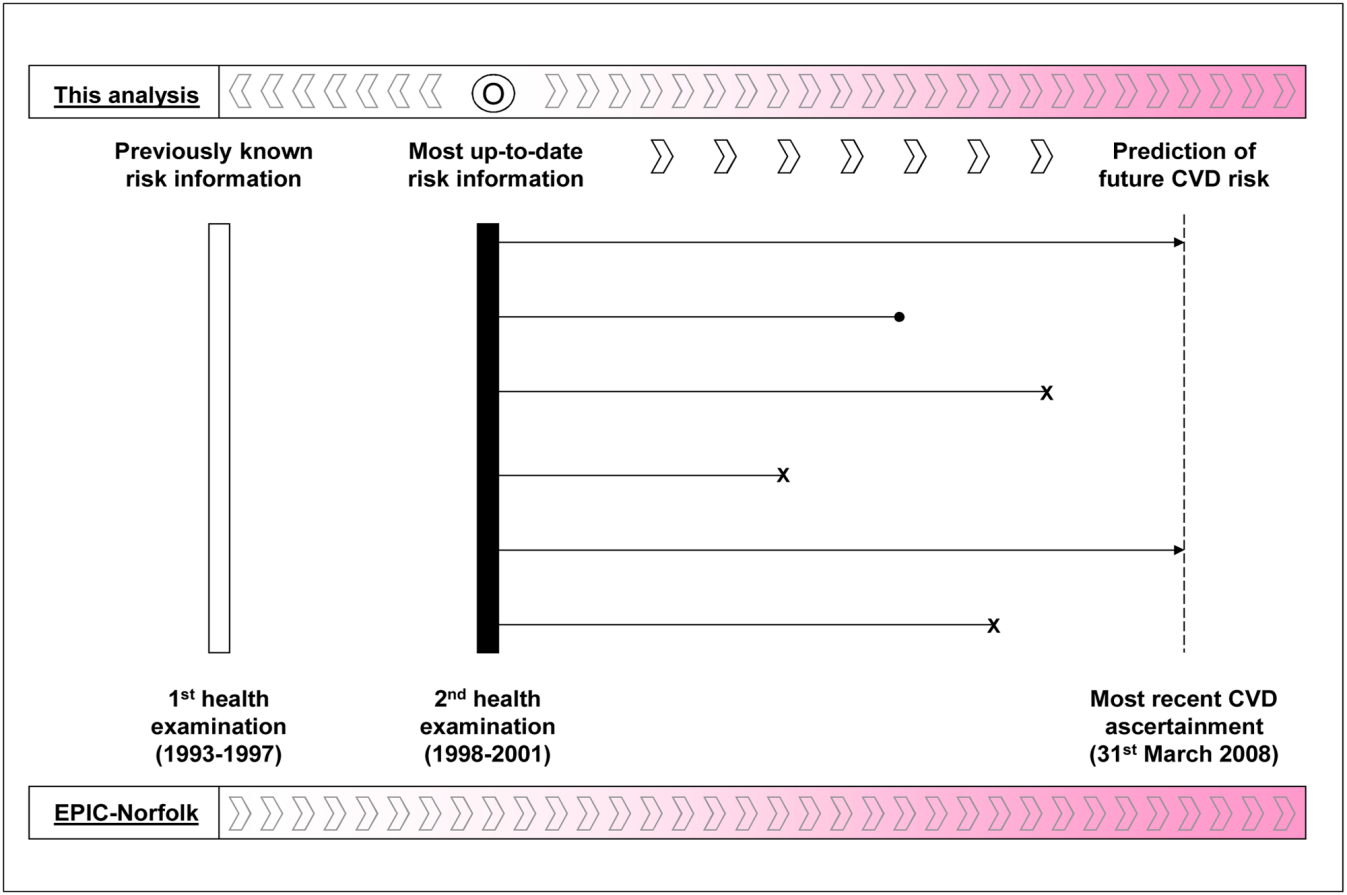
Pictorial diagram of timeframe and information used for investigating the prediction of CVD events in this analysis. x = censored due to diagnosis of cardiovascular disease: • = censored due to death from diseases other than cardiovascular disease.

We defined the second health examination as the point at which the most up-to-date risk information was available (‘index’ assessment) and baseline health examination as providing previously assessed risk information (‘prior’ assessment) (Fig 1). We limited our analysis to individuals with complete data for calculating the Framingham risk score at the first and second health examinations (n = 13,017). We excluded those with prior CVD at the first examination and those who developed a first CVD event up to the second health examination (n = 820), leaving 12,197 individuals for our main analyses. Among these, 390 individuals were prescribed lipid-lowering drugs at baseline or the second health examination. The study was approved by the Norwich District Health Authority Ethics Committee. All participants gave signed informed consent.

### Assessment of cardiovascular risk

We used the most recent Framingham risk equations[17] to quantify the estimated 10-year absolute cardiovascular risk for each individual at the first and second health examinations. Variables used for calculating the Framingham risk score include age, sex, smoking status, prevalent diabetes, use of anti-hypertensive drugs, systolic blood pressure, and total and HDL cholesterol.

### Follow-up and ascertainment of cardiovascular disease

We followed up participants who were free from cardiovascular disease at the time of their second health examination for the development of a first CVD event or death. We report results for follow-up from the second health examination to March 31^st^ 2008, a median of 8.5 years (interquartile range 7.9–9.3). Incident CVD was defined as a composite of fatal or non-fatal cardiovascular disease, including hospitalisation from coronary heart disease and stroke, or death from coronary heart disease, stroke and peripheral vascular disease. This was different from the definition used in the original Framingham study which also includes less severe cardiovascular events (coronary insufficiency, angina, transient ischemic attack, intermittent claudication and heart failure). Vital status for all EPIC-Norfolk participants was obtained via death certification at the Office for National Statistics. Participants admitted to a hospital were identified by their National Health Service number. Hospitals were linked to the East Norfolk Health Authority database, which identifies all hospital contacts throughout England and Wales for Norfolk residents. Hospital record data and vital status information were complete for 95% and 99% of participants respectively. Previous validation studies in this prospective cohort indicated high specificity of such case ascertainment.[18]

### Statistical analyses

We calculated the CVD event rates for groups characterised by (i) different magnitudes of change in the Framingham risk score over time from the first to second health examination and (ii) different levels of the Framingham risk score at the first and second health examinations (absolute value cut-off: <10.0%, 10.0–19.9% and ≥20%).

We compared the discrimination (the area under the receiver operating characteristic curve, aROC), calibration (Hosmer-Lemeshow chi-square statistic), the goodness of fit after cox regression (Gronnesby and Borgan test) and the global model fits (Bayesian information criteria, BIC; and Akaike information criteria, AIC) between the Framingham risk score at the prior and index health examinations (FRS1 and FRS2) and change in the risk score (deltaFRS) for prediction of cardiovascular events.

To investigate the added value of using information from more than one risk assessment, we developed two further Cox regression models (i) using the Framingham risk score at the first health examination and change in the score between the first and second health examination as covariates (FRS1delta) and (ii) using the mean value of the Framingham risk score at both health examinations as covariates (meanFRS). We then computed the measures of predictive ability, as described above, comparing combined information from the two risk assessments (the two new models: FRS21 and FRS2delta) with risk information from the single most up-to-date or index risk assessment (i.e. the Framingham risk score at the second health examination, FRS2). The added value of risk scores computed at two different time points can be seen as the added value of using risk estimates from a previous examination over and above the current risk estimates or vise versa.

To determine the effect of increasing age on the CVD risk estimates and their ability to predict future CVD, we calculated a new FRS2 using risk factor values at the second health assessment but age at the first health assessment, and compared its predictive ability with FRS1 and FRS2 computed by a conventional method.

To determine whether the results differed by the length of time between the two risk assessments, we compared the difference in the predictive ability (aROC) of the Framingham risk score at a single time point (FRS2) and the use of the risk score from a previous examination in addition to the most recent one (FRS21 or FRS2delta). We compared the predictive ability in individuals whose time from the first to second health examination was below and above a median value of 3.7 years.

To demonstrate the benefits of using estimated risk information from repeat risk assessment in clinical practice, we calculated the net reclassification improvement (NRI) to determine whether the most recent risk score (FRS2) helped improve risk classification by a previously assessed risk score (FRS1).

We also performed a subgroup analysis in 11,807 individuals who were not prescribed lipid-lowering drugs up to the time they attended the second health examination in order to examine whether the main results would be influenced by the use of lipid-lowering drugs.

## Results

Characteristics of participants by levels of absolute CVD risk estimated at the first health examination are shown in Table 1. The average 10-year absolute CVD risk increased over time (from 15.5% to 17.5% over a median of 4 years from the prior to the index health examination, p<0.001). Although an increase in absolute CVD risk over time was small, it was largely explained by increasing age. After categorising participants into low, intermediate and high risk at the two health examinations (estimated 10-year risk of <10.0%, 10.0–19.9% and ≥20.0% respectively), we found that two-thirds of the participants remained in the same risk categories at the first and second health examinations.

**Table 1:** Characteristics of EPIC-Norfolk participants included in this analysis by levels of estimated 10-year absolute cardiovascular risk, the Framingham risk score (FRS), at the baseline health examination (n = 12,197).

	Total	Estimated 10-year absolute cardiovascular risk at baseline			p-value *
		<10.0%	10.0–19.9%	≥20.0%	
Number	12,197	5,249	3,655	3,293	
Age, years	58.0 (8.9)	52.0 (6.7)	59.8 (7.4)	65.8 (6.3)	<0.001
Male sex	5,151 (42.2)	1,040 (19.8)	1,688 (46.2)	2,423 (73.6)	<0.001
Social class I-IIIa ^†^	7,641 (62.6)	3,371 (64.2)	2,281 (62.4)	1,989 (60.4)	0.002
Current smokers	1,110 (9.1)	315 (6.0)	298 (8.2)	497 (15.1)	<0.001
Family history of cardiovascular disease^‡^	6,288 (51.6)	2,475 (47.2)	1,990 (54.4)	1,823 (55.4)	<0.001
Body mass index, kg/m^2^	25.9 (3.6)	25.0 (3.6)	26.3 (3.6)	26.8 (3.4)	<0.001
Obesity (body mass index ≥30 kg/m^2^)	1,477 (12.1)	472 (9.0)	504 (13.8)	508 (15.4)	<0.001
Systolic BP, mmHg	133.8 (17.6)	123.1 (12.8)	136.6 (13.9)	147.9 (16.9)	<0.001
Total cholesterol, mmol/l	6.1 (1.1)	5.7 (1.0)	6.3 (1.1)	6.5 (1.2)	<0.001
HDL cholesterol, mmol/l	1.4 (0.4)	1.6 (0.4)	1.4 (0.4)	1.2 (0.3)	<0.001
Triglyceride, mmol/l	1.5 (1.0–2.1)	1.1 (0.8–1.6)	1.6 (1.2–2.2)	1.9 (1.4–2.6)	<0.001
HbA_1c_, % ^§^	5.3 (0.7)	5.1 (0.6)	5.3 (0.7)	5.6 (1.0)	<0.001
Diabetes ^¶^	364 (3.0)	37 (0.7)	71 (1.9)	256 (7.8)	<0.001
Use of anti-hypertensive drugs	1,691 (13.9)	256 (4.9)	478 (13.1)	957 (29.1)	<0.001
Use of lipid-lowering drugs	122 (1.0)	29 (0.6)	51 (1.4)	42 (1.3)	<0.001
FRS at baseline, % over 10 years	11.8 (6.0–21.0)	5.6 (3.7–7.7)	14.1 (11.9–16.7)	29.3 (24.0–37.7)	<0.001
FRS at 2^nd^ health examination, % over 10 years	13.5 (7.3–23.9)	6.7 (4.4–9.6)	16.2 (12.6–20.8)	31.7 (24.5–41.6)	<0.001

Data are presented in number (%), mean (standard deviation) and median (interquartile range) for categorical, normally and non-normally distributed continuous variables respectively.

* differences between groups using *x*^2^ tests for categorical variables, and analysis of variance (ANOVA) or Kruskal-Wallis tests for normally or non-normally distributed continuous variables.

^†^ Registrar General's Social Class: class I = Professional, etc. occupations, II = Managerial and Technical occupations, IIIa = Skilled occupations (non-manual), IIIb = Skilled occupations (manual), IV = Partly-skilled occupations, V = Unskilled occupations.

^‡^ Family history of cardiovascular disease defined as a history of cardiovascular disease in first degree relatives.

^§^ data for only 5,648 individuals with HbA_1c_

^¶^ Diabetes defined as self-report of physician diagnosed diabetes and/or HbA_1c_ at first health examination of ≥6.5%.

### Incidence of cardiovascular events

There were 1,371 cardiovascular events over 99,654 person-years of follow-up, an incidence of 13.8 per 1,000 person-years. Individuals with a higher estimated absolute risk at baseline had a higher incidence rate of cardiovascular events than those with a lower estimated absolute risk. This was also observed for the second health examination. Individuals who had a small change in their estimated cardiovascular risk (< 5%) over time had a low incidence of cardiovascular events in the eight following years (9.8, 95% confidence interval 9.1–10.6 per 1,000 person-years). Those with a big change in their estimated cardiovascular risk (≥5%), either negative or positive change, had significantly high incidence rate of cardiovascular events (21.3; 95%CI 19.4–23.3 and 30.0; 95% CI 26.5–34.7 per 1,000 person-years for those with increased and decreased risk of ≥5%, respectively).

**Fig 2:**
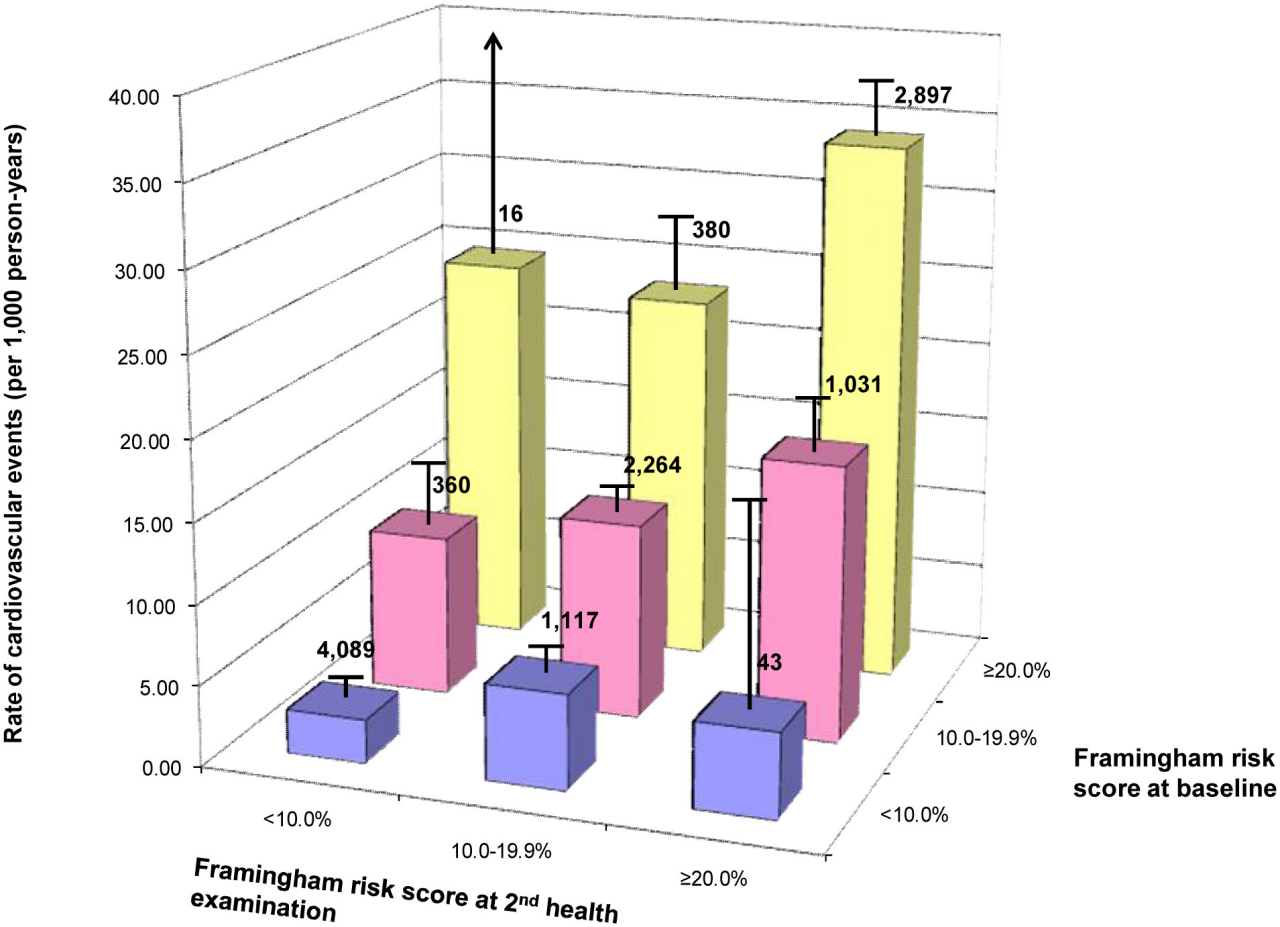
Rates of a first cardiovascular events by levels of estimated absolute risk at the first (FRS1) and second health examination four years later (FRS2). Note: The line above each bar indicates the 95% confidence interval, and the number above each bar represents the number of participants in each risk category.

Individuals with high estimated risk at both health examinations had a higher cardiovascular event rate than those with high estimated risk at only one of the two health examinations (Fig 2). Those with high estimated risk at either health examination had considerably higher rates of cardiovascular events than those who remained in the lowest risk category at both health examinations.

### Prediction of cardiovascular events

The Framingham risk score estimated at the first health examination (FRS1) was slightly better than the score estimated at the second health examination (FRS2) at discriminating between individuals who developed cardiovascular events and those who did not (aROC 0.76 (95%CI 0.75–0.77) and 0.75 (95%CI 0.74–0.76) respectively, p = 0.008), while change in the score over 3.7 years (deltaFRS) provided poor discrimination (aROC 0.53; 95%CI 0.51–0.55) (Table 2). The Framingham risk score at either health examination (FRS1 or FRS2) showed a slightly better model fit than the temporal change risk score. The Framingham risk score was poorly calibrated in the EPIC-Norfolk cohort at both health examinations e.g. cardiovascular risk was over-estimated by 38% and 56% for prior and index health examination respectively.

**Table 2:** Comparisons between the risk scores at different health examinations of the measures of predictive ability for a first cardiovascular event in the EPIC-Norfolk cohort (n = 12,197).

	FRS1	FRS2	deltaFRS	FRS1delta	meanFRS
aROC	0.761 (0.749–0.774)	0.752 (0.739–0.764)	0.528 (0.509–0.547)	0.764 (0.739–0.764)	0.763 (0.751–0.775)
Observed events	1,371	1,371	N/A	N/A	N/A
Predicted events	1,891	2,134	N/A	N/A	N/A
Hosmer-Lemeshow Chi-square statistic	103.6 (p<0.001)	104.0 (p<0.001)	N/A	N/A	N/A
Gronnesby and Borgan test	211.8 (p<0.001)	186.0 (p<0.001)	N/A	N/A	N/A
AIC*	23,972	24,054	24,803	23,953	23,962
BIC*	23,980	24,061	24,811	23,968	23,969

Abbreviation: FRS1 = Framingham risk score at the first health examination, FRS2 Framingham risk score at the second health examination, deltaFRS = change in the Framingham risk score from baseline to the second health examination, FRS1delta = combination of the Framingham risk score at the first health examination and change in the score between the first and second health examination, meanFRS = mean value of the Framingham risk scores from both the first and second health examinations, AIC = Akaike information criteria, and BIC = Bayesian information criteria

* lower values of the AIC and BIC suggest a better model fit.

### Added value of repeat cardiovascular risk assessment

Using estimated absolute risk information from both health examinations (FRS1delta and meanFRS) slightly improved the discriminatory ability of risk information from a single risk assessment (FRS2) (Table 2: aROC 0.76, 0.76 and 0.75 respectively, p<0.001), with a slightly better global model fit.

When replacing age at the second health assessment with age at the first health assessment in the Cox model at the second health assessment, the estimated CVD risk reduced significantly from 17.5% to 15.3% and its predictive ability did not differ from FRS1.

As shown in Table 3, among 1,371 individuals who developed a first CVD event, using information from the most recent risk assessment (FRS2) resulted in a net gain in reclassification of 7.5%, compared to using information from a previous risk assessment (FRS1). In 10,826 individuals who did not experience a CVD event, using information from the most recent risk assessment (FRS2) worsened classification, with a net decline in reclassification of 12.3% compared to using information from the previous risk assessment (FRS1). Therefore, the overall NRI for using information from the most up-to-date risk assessment over that from the previous risk assessment was estimated to be -4.8% (p = 0.99).

**Table 3:** Cardiovascular disease risk classification comparing the Framingham risk score at baseline with the Framingham risk score at the second health examination in the EPIC-Norfolk cohort (n = 12,197).

***Individuals who developed cardiovascular events***				
	Framingham risk score at the 2^nd^ health examination			
	< 10.0%	10.0–19.9%	≥ 20.0%	
Framingham risk score at baseline				**Total**
< 10.0%	95	57	2	**154**
10.0–19.9%	30	230	145	**405**
≥ 20.0%	3	68	741	**812**
**Total**	**128**	**355**	**888**	**1,371**
***Individuals who did not develop cardiovascular events***				
	Framingham risk score at the 2^nd^ health examination			
	< 10.0%	10.0–19.9%	≥ 20.0%	
Framingham risk score at baseline				**Total**
< 10.0%	3,994	1,060	41	**5,095**
10.0–19.9%	330	2,034	886	**3,250**
≥ 20.0%	13	312	2,156	**2,481**
**Total**	**4,337**	**3,406**	**3,083**	**10,826**

Note: The net reclassification improvement was 4.8%, p = 0.99

Larger differences in the predictive ability of the Framingham risk score at a single time point (FRS2) and use of risk scores from previous examination in addition to the most recent one (FRS21 or FRS2delta) were observed for participants with a longer period from the first to second health examination (time period < 3.7 years, aROC for FRS2 and FRS21 0.77 and 0.78 respectively, *X*^2^ = 6.40, p = 0.011; corresponding estimates for time period ≥3.7 years, 0.73 and 0.75, *X*^2^ = 21.37, p<0.001).

In a subgroup analysis in 11,807 individuals who were not prescribed lipid-lowering drugs, we found similar results e.g. using information from repeat risk assessments modestly improved the predictive ability of information from a single risk assessment.

## Discussion

Using data from a large population-based prospective cohort with repeat health examinations, we estimated the predictive ability of approaches for estimating risk using information from a single contemporary risk assessment and incorporation of risk information collected previously. Compared to risk information from a single risk assessment, using information from repeat risk assessments over a period of 4 years slightly improved discrimination but did not improve risk classification.

### Comparison with previous studies

Limited evidence exists concerning the optimal frequency of risk assessment in asymptomatic individuals. The National Cholesterol Education Program Adult Treatment Panel (NCEP-ATP III) guideline states that the Framingham equations are not intended to be used to track changes in risk over time as risk factors are modified as a result of treatment.[5] The NCEP-ATP III, however, suggests measuring lipid profiles at least once every 5 years in low risk adults aged ≥20 years old, but does not provide any clear guidance on how often cardiovascular risk measured by the Framingham equations should be reassessed in apparently healthy individuals who do not receive intensive treatments. The European cardiovascular societies[6] and Joint British Societies[4] guidelines suggest that global risk assessment be repeat every 5 years, without citing any supporting evidence. The recent guideline by the American College of Cardiology Foundation and American Heart Association stresses that research on the optimal timing to begin risk assessment and repeat risk assessment in asymptomatic low-intermediate risk individuals is needed.[10]

Using data from the Framingham Heart Study with repeat risk factor measurements, Karp et al found that the predictive ability of the multivariate risk score estimated using updated data was better than the score estimated previously.[13] They also suggested that the optimal frequency of updating cardiovascular risk information may vary across subpopulations with different ages and sexes, and that updating risk information every two years may not be preferable to updating every six years. However, the authors only examined the discriminatory ability of the models. Our study examined the NRI of different models, which is useful in terms of reclassification of individuals who would develop future cardiovascular events.[14]

A modelling study commissioned by the UK Department of Health (DH) suggested that cardiovascular risk assessment based on the Framingham risk equations or similar tools should be repeated every 5 years in individuals aged 40–74 years. The authors concluded that when followed by appropriate preventive interventions, the risk assessment programme is likely to be cost-effective compared to no screening, and to have the highest net benefits compared to other screening strategies.[19] The authors accounted for changes in levels of risk factors and cardiovascular risk over time in their model.[20] However, they assumed no change in cholesterol over time, that nobody would take up smoking if they did not smoke already and that those who had quit smoking for more than 12 months would not start smoking again. The authors also assumed that a proportion of the population would become hypertensive each year, while blood pressure would be stable among the remainder of the population.[20] Given these limitations, there is continuing uncertainty over recommendations for optimal frequency for cardiovascular risk assessment.

Of note, only a small number of participants were on lipid lowering drugs either at first or second health examination (1993–2001). This might be explained by that the majority of EPIC-Norfolk participants were generally apparently healthy at the time of recruitment. Also, as landmark large-scale trials on benefits of statins on primary and secondary prevention of cardiovascular disease were published in mid-late 1990s (e.g. 4S, LIPID, AFCAPS/TexCAPS, HPS), the use of lipid lowering drugs, particularly statins for primary prevention, was expectedly low by 1997 and probably was slowly increasing during 1998–2001 before the concept of primary prevention of CVD through use of statins was widely accepted.

### Policy implications

Our findings suggest that repeating risk assessments within a period of 4 years modestly improve prediction compared with a single risk assessment. Indeed, using information from repeat risk assessments over such a relatively short time does not seem to accurately reclassify people into appropriate risk categories. A sensitivity analysis examining the predictive ability of the risk score calculated by keeping age constant suggests that changes in CVD risk factors over a short period of four years may contribute little to changes in estimated CVD risk and added predictive values. Accordingly, our findings do not suggest that a new CVD risk assessment is needed to be done, at least within a period of four years. However, our stratified analysis suggests that repeat risk assessment over a longer period may improve the incremental predictive ability over a single risk assessment to a greater extent than repeating assessment over a shorter period. Therefore, value of repeating risk assessment over a longer period of time remains uncertain and merits further investigation. Of note, our analysis was concerned with effects of repeat assessment on prediction of cardiovascular disease. Further research is therefore needed to investigate how often the assessment of individuals’ short-term cardiovascular risk should be repeated to optimise population risk stratification and the delivery of scarce preventive treatment resources to those with most to gain.

Some investigators proposed the use of lifetime risk for cardiovascular disease in conjunction with a shorter-term 10-year risk,[9,21–23] as there is a significant discrepancy between the levels of short-term and lifetime risk.[9,23] This is particularly pronounced in young and middle-aged men who had a low short-term risk, but a high lifetime risk. Stratifying a population using information only on short-term risk may overlook a significant number of people with high lifetime risk, who might benefit from preventive interventions.[9,22] There is also evidence that information on high lifetime risk might lead to more intensive prescription of aspirin or lipid-lowering drugs by primary care physicians.[24] However, there remains uncertainty over the role of lifetime risk estimation in individualised risk management.[25] In deed, it remains unclear whether clinicians should prescribe lipid lowering drugs in those with a low short-term but high lifetime estimated risk. It is unclear if it is more cost-effective to start prescribing lipid-lowering drugs to young adults, who are unlikely to develop cardiovascular disease within the short term, in order to prevent cardiovascular events in the longer term, or to repeat assessments of short-term risk over time and to prescribe treatment when the short-term risk is beyond a 20% risk threshold.[23]

### Strengths and limitations

To our knowledge, our study is the first to investigate the potential use of information from repeat risk assessment over time for prediction and reclassification of cardiovascular risk. We used information on risk factors from repeat health examinations to calculate widely used measures of predictive ability and reclassification for the development of cardiovascular events over a 9-year follow-up period However, four years may not have been sufficiently long to exclude the possibility that repeat risk assessment has some merit. Further research is needed to investigate the impact of approaches using repeat risk assessment over a longer period, e.g. 10 years, on cardiovascular risk prediction and classification in a general population, as well as complementary strategies to improve risk stratification, e.g. a stepwise approach including both short-term and long-term risk.

Our findings should be interpreted in the light of a number of limitations. Due to our research question, we excluded those who did not have information on the second health examination. Those who attended the follow-up examination were healthier than those who did not (according to their baseline risk factor profiles), which might have led to an underestimation of cardiovascular event rates in the whole cohort. However, it is not clear how this may have influenced the predictive ability and reclassification of approaches using information from repeat risk assessments. As only 25% of the cohort were followed from the second health examination for up to 10 years, the incidence of CVD events of the whole cohort may be low. This might have altered the discrimination and calibration of the risk equations in this analysis.

Using hospital linkage data for the ascertainment of CVD outcomes might lead to misclassification of non-fatal CVD events, as not all non-fatal CVD cases lead to hospital admission. However, this method captures the nonfatal events of most clinical importance, and previous validation studies in this cohort indicated high specificity of such case ascertainment.[18] Additionally, as the definitions of cardiovascular events used in the present study and the Framingham study are different, this might have altered the predictive ability of the risk scores. Lastly, as the majority of EPIC-Norfolk participants are of European descent, the generalisability of our findings to other ethnic groups and populations is limited.

### Conclusions

Compared to a single cardiovascular risk assessment, information on repeat risk assessments over 4 years may slightly improve the ability to discriminate individuals according to their cardiovascular risk. However, such an approach does not improve risk reclassification in this British population. Further research is needed to investigate the potential benefits and costs of different frequencies of cardiovascular risk assessment, e.g. 10 years vs. lifetime horizon, and to explore effects of risk assessment and communication on patient and practitioner behaviour.
